# Energy and macronutrient intakes in preschool children in urban areas of Ho Chi Minh City, Vietnam

**DOI:** 10.1186/1471-2431-8-44

**Published:** 2008-10-18

**Authors:** Dieu TT Huynh, Michael J Dibley, David W Sibbritt, Hanh TM Tran

**Affiliations:** 1 Nutrition Centre of Ho Chi Minh City, Ho Chi Minh City, Vietnam; 2 School of Public Health and the George Institute for International Health, University of Sydney, Australia; 3 Centre for Clinical Epidemiology and Biostatistics, Faculty of Health, University of Newcastle, Australia

## Abstract

**Background:**

An increasing prevalence of overweight and obesity has been documented in preschool children in Ho Chi Minh City (HCMC), Vietnam. However, little is known about what preschool children in HCMC eat or how well their nutrient intake meets nutrient recommendations. This study aims to describe the energy and macronutrient intake and compare these nutrient intakes with the recommendations for Vietnamese children aged four to five years.

**Methods:**

The data comes from the baseline measurement of a one year follow-up study on obesity in 670 children attending kindergartens in HCMC. Dietary information for each child at the school and home settings was collected using Food Frequency Questionnaires (FFQs), by interviewing teachers and parents or main caregivers. The average energy and nutrient intake in a day was calculated. The proportion of children with energy intake from macronutrients meeting or exceeding the recommendations was estimated based on the 2006 recommended daily allowance (RDA) for Vietnamese children in this age group.

**Results:**

The dietary intake of the participants contained more energy from protein and fat, particularly animal protein and fat, and less energy from carbohydrates, than the RDA. Most children (98.1%) had mean energy intake from protein greater than the recommended level of 15%, and no child obtained energy from animal fat that was in accordance with the recommendation of less than 30% of the total fat intake. Nearly one half of children (46.5%) consumed less than the advised range of mean energy intake from carbohydrate (60%–70%).

**Conclusion:**

In this preschool child population in HCMC, in which obesity is emerging as major public health problem, there is an imbalance in dietary intake. Healthy eating programs need to be developed as a part of an obesity prevention program for young children in HCMC.

## Background

It is known that young children have high nutritional demands and that a balanced diet in childhood is very important to ensure optimum growth and development [1]. Recently, there has been growing evidence that childhood diet may have important implications for the development of chronic disease in later life [2-5]. Studies examining the early natural history of heart disease have demonstrated the relationship between early dietary habits, such as high intake of fat and energy, and the subsequent development of cardiovascular disease [2,3]. Several longitudinal studies have found an association between early protein intake and changes in body fatness later in life, generating an hypothesis that high levels of protein consumption in early childhood may lead to the later development of obesity [4,5]. The assessment of dietary intake is therefore essential for monitoring the nutritional status of preschool child populations, as well as for conducting epidemiologic research on the association between diet and health in this age group [6].

The economic situation in Vietnam during the past two decades has improved considerably, especially in Ho Chi Minh City (HCMC), which is the economic and trade centre for southern Vietnam and has the highest rate of economic development in the country [7]. The effects of rapid economic development on the nutritional status of the urban adolescent and adult populations in HCMC are similar to that seen in other developing countries undergoing a "nutrition transition" [8,9].

Although there is evidence that the preschool child population in the urban areas of HCMC is also undergoing a "nutrition transition" [10], little is known about what these children eat or how well their dietary intake meets nutrient recommendations. An assessment of dietary intake in this young child population, which appears to be experiencing an obesity epidemic with the prevalence of overweight of 20.5% and obesity of 16.3% according to International Taskforces definition [10], would provide important information to help understand the high prevalence of childhood obesity. This study aimed to describe the energy and macronutrient intake of preschool children aged 4 to 5 years, using the baseline data of a cohort study, and compares these nutrient intakes with the recommendations for Vietnamese children of this age.

## Methods

### Subjects

This study was conducted in March, 2005, and was the baseline measurement for a one-year cohort study [10]. The baseline survey used two-stage cluster sampling with stratification by groups of districts (wealthy and less wealthy districts) in the first stage. Twenty preschools were selected in the first stage using a proportionate to school population size (PPS) method based on a sampling frame of all preschools in urban areas of HCMC. In each selected school, a list of children aged 4 to 5 years was prepared and 35 children were selected by systematic random sampling. There were 670 children aged 4 to 5 years, and their parents, who participated in this study.

Both the Human Research Ethics Committee of the University of Newcastle, Australia and Department of Health in HCMC approved the procedures used in the study. The parents participating in the study signed consent forms before the data collection began.

### Dietary measurement

Dietary intake was measured using a food frequency questionnaire (FFQ) adapted from one that has been validated for preschool children aged 6 to 36 months in HCMC (Unpublished report). The original validation study aimed to evaluate the reliability and validity of the FFQ assessing nutrient intake in preschool children aged 6 to 36 months in sub-urban areas of HCMC in 2003. The preliminary food list was created from foods reported in a survey of preschool aged children using 24 hour recalls. Other foods were identified from the food composition table and focus group discussion. The findings showed good short-term (one month apart) and adequate long-term (four month apart) reproducibility. For example, correlation coefficients for energy and protein in short-term reproducibility were 0.70 and 0.73 respectively, and in long-term reproducibility were 0.51 and 0.57 respectively. The validity study was conducted using six 24 h-recalls as a reference method. The results also revealed that the FFQ had high validity, especially for protein (r = 0.41), zinc (r = 0.52) and phytate (r = 0.48) intakes.

In the present study, the food list in the available FFQ was modified using information collected through 24 hour recalls from forty eight children aged 4 to 5 years in urban areas of HCMC. Foods reported in the 24 hour recalls that were not on the food list were considered for inclusion on the food list. These foods were either grouped with existing foods on the list or were added as new foods on the list. Foods on the FFQ food list that were not reported in the 24 hour recalls were also revised. Foods were removed from the list if they were mainly baby foods such as breast milk or infant formula. The portion size of food items in the FFQ was adjusted by asking twelve mothers with children of similar age to identify the usual portion sizes served to their children. For the 89 food items in this FFQ, the usual frequency of consumption of each food was recorded and the portion sizes were estimated with the help of visual aids (a real size picture book of portion sizes of different foods). Both preschool teachers and parents were asked to assess the child's dietary intakes at school and home using the FFQ (see Additional file 1).

The interviews were conducted by staff of the Community Nutrition Department of the Nutrition Centre, HCMC, who had experience in obtaining dietary information by interview. They were trained in interviewing techniques and specifically how to use the FFQ.

Energy intake and macronutrients per day for each child were calculated using the following formula:

[Reported daily frequency of consumption] * [Reported portion size for each food item] * [Standard portion size in grams of that food item] * [Nutrients per 100 gram]

The nutrient values per 100 grams of food were obtained from the Vietnam National Food Composition Tables and the Vietnam Nutrients and Composite Foods Database Software (Eiyokun-Nutrition Centre HCMC) [11]. Energy and macronutrient intake for each food item was then totalled to obtain the total intake score per day for each subject.

The children have breakfast, lunch, and snacks at preschool and dinner at home during five weekdays. They eat all meals at home during two weekend days. The average macronutrient intake in a day was calculated to take into account the food intake at the kindergarten over five school days and at home over seven days, using the following formula:

Average amount of each nutrient = [teacher reported nutrient + parental reported nutrient] * 5/7 + [parental reported nutrient * 2/7]

The energy conversion factors used for protein, fat and carbohydrate were 4 kcal/g, 9 kcal/g and 4 kcal/g respectively. The energy from each macronutrient energy source was divided by the total energy intake and multiplied by 100 to produce the percentage contribution. The energy intake data was checked to make sure that no participant reported an implausible energy intake (<500 kcal per day or >5000 kcal per day) [12]. It was not possible to apply methods for estimating underreporting of nutrient intakes because these methods have been developed for use with older children and adults [13]. In addition, FFQs are only suitable for ranking subjects rather than estimating the level of nutrient intake [14].

The proportion of children with total caloric intake and energy intake from macronutrients meeting or exceeding the recommendations was estimated based on the 2006 RDA for Vietnamese children in this age group [15]. The RDA for total energy intake for children of both genders in this age is 1470 kcal. The recommended ranges for the percentage of energy from protein, fat and carbohydrate are 12% to 15% (50% animal protein), 18% to 25% (70% vegetable fat: 30% animal fat) and 61% to 70%, respectively [15].

According to the Joint WHO/FAO Expert Consultation, free sugars' refers to all monosaccharides and disaccharides added to foods by the manufacturer, cook or consumer [16]. There are no recommendations on free sugar consumption in children. To estimate the number of children who met or exceeded the recommended intake of free sugar, we have used the recommendations from the 2002 Joint WHO/FAO Expert Consultation for free sugar, which defines a population goal for free sugar intake of less than 10% of total energy [16].

Information on 17 household assets including household vehicles, entertainment appliances, and household appliances was used to assess household wealth status. An index of these household assets was constructed using an established principal component analysis method [17] to weight the contribution of each asset to the index. Once calculated the wealth index was used to categorize subjects into tertiles. The highest tertile of wealth index represented the richest group and the lowest wealth index tertile represented the poorest group.

### Statistical analyses

Statistical analyses were conducted using STATA version 9.0 (2005, Stata Corporation, College Station, TX, USA). The Stata "svyset" commands were used to adjust the analyses for the stratified two-stage cluster sampling design using Taylor linearized variance estimation. Sampling weights for each stratum were calculated for use with the "svyset" commands. All nutrients were checked for normality. Group means and 95% confidence intervals for each nutrient by gender and household wealth index were calculated. Student's t-test was used to compare two groups of normally distributed continuous data. The one-way ANOVA, with Bonferroni correction were employed for comparison of three or more group means of normally and non-normally distributed continuous data, respectively.

## Results

### Characteristics of the subjects

In the baseline study, parents/caregivers and teachers of 670 children completed the FFQ interviews. No participant was excluded because of implausible reported energy intake. The response rate for this study was 95.7%.

Table 1 presents the characteristics of children participating in this study. The proportion of boys (49.6%) and girls (50.4%) in the study was similar. Children of Kinh ethnicity accounted for 84.9% of the sample and the remaining 15.1% were Chinese. There was no gender difference across three group of wealth household index.

**Table 1:** Characteristics of sample children

**Characteristic**	**Boys (N = 333)**		**Girls (N = 337)**		**Total (N = 670)**	
	
	**%**	**95% CI**	**%**	**95% CI**	**%**	**95% CI**
**Age in months**						
48–53	37.9	30.2, 46.3	33.0	28.5, 37.8	35.4	31.1, 40.0
54–59	42.5	35.1, 50.3	46.5	40.8, 52.4	44.5	39.4, 50.0
60–63	19.6	15.9, 23.9	20.5	15.5, 26.6	20.1	17.0, 23.5
**Ethnicity**						
Vietnamese	85.1	71.4, 92.9	82.8	72.3, 89.9	83.9	72.3, 91.2
Chinese	14.9	7.1, 28.7	17.2	10.1, 27.7	16.1	8.7, 27.7
**Household wealth**						
Poorest	33.6	27.9, 41.1	33.5	23.6, 54.1	225	26.9, 41.6
Middle	30.3	25.7, 35.3	35.9	29.5, 44.2	222	29.1, 38.1
Wealthiest	36.1	29.7, 41.8	30.6	22.5, 38.8	223	26.8, 39.3

### Energy and macronutrient intakes

Table 2 shows the description of macronutrient intake in absolute values and the percentage of energy from macronutrients, in boys and girls combined and separately. Energy intake and macronutrient intake were normally distributed. In general, there was a gender difference in macronutrient intake, where boys were observed to have higher nutrient intake than girls. The intake of protein, fat and carbohydrates including added sugar was greater for boys than for girls, although the p-value for fat was only of borderline significance (p = 0.052). Total energy intake for boys was higher than for girls (p = 0.0047). Boys consumed more animal and vegetable protein than girls (p = 0.0256 and p = 0.0180 respectively), whilst the higher fat consumption in boys came from animal fat rather than vegetable fat (p = 0.056). However, the percentage of energy from protein, fat and carbohydrates were not significantly different between boys and girls.

**Table 2:** Mean energy intake, nutrient intakes and the percentage of energy from macronutrients by gender in preschool children

**Nutrients**	**Total (n = 670)**		**Boys (n = 333)**		**Girls (n = 337)**		**p-value**^1^
	
	**Mean**	**95% CI**	**Mean**	**95% CI**	**Mean**	**95% CI**	
**Energy (kcal) Protein**	1602.0	1571.0, 1635.0	1647.0	1598.0, 1695.0	1560.0	1526.0, 1593.0	0.0047
Grams	74.0	72.6, 75.2	75.7	73.9, 77.5	72.1	70.4, 73.8	0.0117
% kcal	18.5	18.2, 18.7	18.4	18.1, 18.7	18.5	18.2, 18.7	0.8417
**Animal protein**							
Grams	47.9	46.7, 49.0	49.0	47.6, 50.4	46.7	45.3, 48.1	0.0256
% kcal	12.0	11.7, 12.2	12.0	11.6, 12.3	12.0	11.6, 12.3	0.7320
**Vegetable protein**							
Grams	25.8	25.1, 26.5	26.5	25.5, 27.4	25.1	24.2, 25.9	0.0180
% kcal	6.4	6.3, 6.5	6.4	6.3, 6.5	6.4	6.3, 6.6	0.6550
**Fat**							
Grams	38.4	37.1, 39.6	39.3	37.6, 40.9	37.5	36.3, 38.6	0.0516
% kcal	21.4	20.9, 21.9	21.3	20.8, 21.9	21.4	20.8, 22.0	0.7496
**Animal fat**							
Grams	26.4	25.3, 27.5	27.0	25.7, 28.3	25.8	24.7, 26.9	0.0560
% kcal	14.8	14.2, 15.4	14.8	14.2, 15.3	14.8	14.1, 15.5	0.8670
**Vegetable fat**							
Grams	11.8	11.3, 12.3	12.1	11.3, 12.8	11.5	10.8, 12.2	0.1830
% kcal	6.5	6.2, 6.7	6.5	6.2, 6.8	6.5	6.2, 6.8	0.8400
**Carbohydrate**							
Grams	241.1	235.5, 246.6	248.2	240.2, 256.1	234.0	227.8, 240.3	0.0026
% kcal	60.3	59.7, 60.9	60.3	59.6, 61.1	60.3	59.6, 61.0	0.8850
**Free sugar**							
Grams	25.5	24.3, 26.7	26.9	25.7, 28.1	24.2	22.2, 26.2	0.0030
% kcal	6.3	6.0, 6.6	6.4	6.2, 6.7	6.1	5.6, 6.7	0.1029

^1 ^p-value was calculated from Student's t-test

The energy intake and macronutrient intake of children by gender and the household wealth index are shown in Table 3. Total energy intake across the three groups of wealth status was not significantly different. However, for boys, a greater intake of protein, especially animal protein, was found in those from the wealthiest families compared with their peers from the poorest households (p = 0.006). Such a relationship was not seen for protein intake in children of both genders combined or in girls. Children from the wealthiest families appeared to consume more animal fat than those from the poorest households (p = 0.06), although the total fat intake (including animal and vegetable fat) was not significantly different between the groups. This difference came mainly from a significantly higher intake of animal fat in boys rather than girls, from the wealthiest families (p = 0.008). There were no significant differences in the consumption of free sugar across the three household wealth index groups.

**Table 3:** Mean energy intake and macronutrient intakes in preschool children by gender and the household wealth status

**Nutrients**		**Poorest (n = 225)**		**Medium (n = 222)**		**Wealthiest (n = 223)**		**p-value**
		
		**Mean**	**95% CI**	**Mean**	**95% CI**	**Mean**	**95% CI**	
**Energy (kcal)**	Total	1592.2	1541.8, 1642.6	1598.8	1555.0, 1642.7	1617.8	1578.3, 1657.2	0.555
	Boys	1583.1	1509.0, 1657.4	1677.2	1590.6, 1763.9	1681.3	1622.0, 1740.6	0.106
	Girls	1601.3	1540.0, 1662.6	1534.6	1484.5, 1584.7	1543.4	1492.0, 1594.9	0.532
**Protein (g)**	Total	72.5	70.4, 74.6	76.3	72.6, 80.1	76.1	74.3, 77.9	0.070
	Boys	71.4	68.2, 74.5	70.5	67.6, 73.4	79.3	77.1, 81.6	0.006^1^
	Girls	73.6	70.0, 77.3	73.1	71.0, 75.3	72.3	69.9, 74.7	0.308
**Animal protein (g)**	Total	25.9	24.8, 27.0	47.2	45.4, 49.0	50.1	48.7, 51.5	0.008^1^
	Boys	25.7	24.2, 27.3	48.9	46.1, 51.7	52.6	50.9, 54.2	0.000^1^
	Girls	51.3	48.0, 54.5	45.8	43.4, 48.3	51.2	49.1, 53.3	0.270
**Vegetable protein (g)**	Total	28.1	26.9, 29.3	25.7	24.9, 26.4	25.8	24.8, 26.8	0.931
	Boys	27.9	26.2, 29.6	27.2	25.9, 28.5	26.6	25.2, 27.9	0.405
	Girls	26.1	24.9, 27.3	24.4	23.5, 25.3	24.9	23.6, 26.2	0.633
**Fat (g)**	Total	37.8	36.3, 39.2	38.5	36.6, 40.4	38.8	37.5, 40.0	0.581
	Boys	37.4	34.9, 39.8	39.8	36.6, 43.0	40.6	38.9, 42.4	0.099
	Girls	38.2	36.0, 40.3	37.5	35.6, 39.4	36.6	35.0, 38.3	0.786
**Animal fat (g)**	Total	25.4	24.3, 26.4	26.5	24.9, 28.2	27.3	26.1, 28.5	0.060
	Boys	25.2	23.7, 26.8	27.0	24.5, 29.5	28.7	27.5, 30.0	0.008^1^
	Girls	25.5	23.7, 27.3	26.1	24.5, 27.8	25.6	24.1, 27.1	0.966
**Vegetable fat (g)**	Total	12.2	11.5, 12.9	11.8	11.2, 12.4	11.3	10.8, 11.9	0.279
	Boys	12.0	10.9, 13.0	12.6	11.4, 13.7	11.8	10.8, 12.7	0.454
	Girls	12.4	11.5, 13.3	11.2	10.3, 12.1	10.9	10.2, 11.5	0.223
**Carbohydrate (g)**	Total	240.8	229.6, 252.1	240.4	234.3, 246.6	241.7	234.3, 249.0	0.803
	Boys	241.3	232.5, 250.1	254.0	241.3, 266.8	250.2	240.1, 260.3	0.248
	Girls	241.1	232.6, 249.5	229.3	222.8, 235.8	231.7	221.9, 241.4	0.687
**Free sugar (g)**	Total	25.9	23.6, 28.1	26.1	24.7, 27.61	24.5	22.6, 26.5	0.659
	Boys	25.9	23.6, 28.2	25.6	26.3, 30.9	26.4	24.3, 28.4	0.199
	Girls	25.8	22.8, 28.8	24.1	22.0, 26.2	22.4	20.2, 24.7	0.547

^1 ^Means are significantly different between poorest and wealthiest groups, based on ANOVA Bonferroni procedure

### Comparison of nutrient intakes with the RDA

Table 4 shows the percentage of children who had a percentage of energy from macronutrients that met or exceeded the 2006 RDA for children at this age. More than one half of the children had a total caloric intake greater than recommended (58.3%). There were more boys with total energy intake exceeding the recommended level than girls (p = 0.037). There was no child with mean energy intake from protein, expressed as a percentage of energy from protein, less than 12% (RDA: 12–15%). There were only 2% of children who met the recommendation while the other 98% exceeded the recommendation for energy intake from protein. Additionally, the energy contribution from animal protein was found to be higher than the recommendation in all children (>50% of total energy intake from protein).

**Table 4:** Percentage of preschool children with percent of energy from macronutrients meeting or exceeding the 2006 RDA by gender

**Energy intake and nutrients**	**Total (n = 670)**		**Boys (n = 333)**		**Girls (n = 337)**		**p-value**^1^
	
	**%**	**95% CI**	**%**	**95% CI**	**%**	**95% CI**	
**Energy (kcal)** meeting RDA	31.7	27.5, 36.3	29.3	24.7, 34.4	34.1	28.6, 40.1	0.186
**Energy (kcal)** exceeding RDA	58.3	54.6, 62.0	62.5	57.3, 67.5	54.2	49.4, 58.9	0.037
**% energy from protein** meeting RDA	1.9	0.9, 3.8	2.7	1.1, 6.6	1.0	0.4, 2.7	0.236
**% energy from protein** exceeding RDA	98.1	96.2, 99.1	97.3	93.4, 98.9	99.0	97.3, 99.6	0.236
**% energy from animal protein** exceeding RDA	100.0	_	100.0	_	100.0	_	_
**% energy from fat** meeting RDA	86.0	81.4, 89.7	85.2	80.2, 89.1	86.8	80.5, 91.4	0.594
**% energy from fat** exceeding RDA	12.7	8.9, 18.0	12.6	7.8, 19.8	12.9	8.2, 19.6	0.950
**% energy from animal fat** exceeding RDA	100.0	_	100.0	_	100.0	_	_
**% energy from carbohydrate** meeting RDA	52.9	46.7, 59.1	51.5	44.2, 58.7	54.3	45.2, 63.1	0.649
**% energy from carbohydrate** exceeding RDA	0.6	0.2, 2.3	0.8	0.1, 5.4	0.4	0.1, 1.6	0.990
**% energy from free sugar** exceeding recommendation^2^	5.3	3.6, 7.8	5.5	3.6, 8.3	5.2	2.7, 9.5	0.592

^1 ^p-value was calculated from Student's t-test

^2 ^Based on the recommendation on free sugar from the Joint WHO/FAO Expert Consultation 2002

Compared with protein intake, there was a higher proportion of children (73%) with mean energy intake from fat meeting the recommendation (RDA: 18–25%) while the other 12.7% exceeded the recommended level for energy intake from fat. Similarly to energy consumption from animal protein, the energy contribution from animal fat in all children was greater than the recommended 30% of total energy intake from fat. The dietary intake of 53% of the children met the recommended percentage of energy from carbohydrates and only 0.6% exceeded the recommended range. Thus, nearly half the children consumed less carbohydrate than recommended. Consumption of free sugar in excess of the recommendation was only observed in 5.3% of the children.

## Discussion

### Main findings from the study

In this study, the dietary intake of children aged 4 to 5 years from urban areas of HCMC was measured by combining the responses to an FFQ administered to both the teacher and parents of the children. The diet of this child population contained more energy from protein and fat, and less energy from carbohydrate, than recommended. Such dietary patterns appear to be more pronounced in boys rather than in girls and in children from wealthier families.

### Comparison with other studies

An increasing prevalence of overweight and obesity among preschool aged children in urban areas of HCMC has been reported recently [10,18]. Therefore, the mean energy and macronutrient intake of this population are of great interest. While studies on dietary patterns in young children in developed countries have observed a slight decrease or stagnation in total energy intake in young children over past decades [1,19], the findings in the present study have shown that more than half the children aged 4 to 5 years (58.3%) consumed total energy intake exceeding the recommended level for children of this age. However, there are no previous reports of dietary intake for comparison of the findings from this study and for assessing how dietary intake has changed recently.

There was an imbalance in the protein intake among children in the study, with higher than expected protein intake and a higher contribution of protein to energy intake than recommended. Almost all children (98.1%) had a diet with a percentage of energy intake from protein that exceeded the recommended range of 12–15%. Mean energy intake from protein for children in this study was greater than that reported for English children aged seven years (13.0%) [1], Italian children aged five years (15%) [20], and Belgium children aged 4 to 6.5 years (15.5% for boys and 15.1% for girls) [21]. It has been hypothesised that a high protein intake may trigger adipocyte multiplication by stimulating the production of an insulin-like hormone [4]. High protein consumption in preschool children in this study indicates that this dietary factor needs to be considered as a candidate risk factor for longitudinal changes in adiposity in this population.

Compared with mean intake from protein, a lower percentage (13%) of the children consumed energy from fat that exceeded the recommended levels. Thus, along with high consumption of protein, children also had high intakes of fat, which may be another possible factor influencing the increasing prevalence of obesity in this young child population. Moreover, the consumption of animal fat contributed 70% to the overall percentage of energy from fat, which far exceeded the recommended level of 30%. The reported mean fat intake, as a percentage of energy from fat (21.4%), was much lower in children of a similar age in the United States (31.2%) [22], Italy (35.3% for boys and 36.1% for girls) [20], and Belgium (30.1% for boys and 29.7% for girls) [21]. Nevertheless, animal fat is the source of saturated fats that are associated with cardiovascular diseases in children and adults when high levels are consumed [3,23]. These results imply an early exposure to a risk factor for the development of an important chronic disease later in life.

Nearly one half of the children (46.5%) had a contribution of carbohydrates to their total energy intake below the recommended levels. This finding suggests that energy from carbohydrate has been replaced by protein and fat intake. This pattern indicates a shift away from traditional Vietnamese dietary patterns, where rice (carbohydrate) is the main source of calories. Although dietary intake data for children at the specific ages examined in this study are not available at the national level, the findings of this study are consistent with the dietary changes of increasing fat and protein consumption in Vietnamese adults as reported in the national food survey in 2000, especially in urban populations such as HCMC [24].

In our study, only a small percentage of children were found to have an intake of free sugar intake above the recommended level (>10% of total energy intake). The role of sugar in promoting obesity remains controversial since different findings on this association have been reported [25]. Limitations in the methodology, lack of control for confounders and biased reporting of diet are thought to contribute to the inconclusive findings of these studies [16,25]. However, high intake of added sugar is considered to displace other nutrient content, and so restriction of free sugar should be encouraged [16].

In this study, gender differences in nutrient intake were observed, and boys rather than girls were shown to have greater intakes of total energy, protein and fat intake. This gender difference was consistent by household wealth status. The gender difference in dietary intake patterns were seen to match with the significantly higher levels of obesity in boys, as reported recently [10,18]. It may be hypothesised that culturally, boys are preferred over girls and often better nourished than girls in Vietnam, thus resulting in a gender difference in dietary patterns and overweight and obesity prevalence. However, further research is needed to examine this hypothesis.

The household wealth status appears to have an impact on food intake patterns in this child population. Children from wealthiest families had higher intakes of energy, protein, fat and carbohydrate, than their counterparts from poorer households. The role of household wealth status on food habits differed in our study from that reported in developed countries. For example in the United States, lower community and household socioeconomic status and lower parental education levels have been reported as being associated with greater consumption of high energy foods and lower consumption of fruit and vegetables [26,27]. In contrast, in our study, higher socioeconomic class was related to higher total energy and animal fat consumption.

New dietary recommendations for Vietnamese children developed by the National Institute for Nutrition (NIN) and reviewed by the Vietnamese Association of Pediatrics in 2006, have been used in our study. These RDAs have a number of differences compared with the recommendations for other countries, such as the dietary reference intake (DRI) from the Institute of Medicine in USA [28]. For example, the percentage of energy from fat is seen to be lower than recommended in the DRI for the USA (25–35%). Such differences may be explained in the context of Vietnam. From 1980s to late 1990s, the dietary intake of Vietnamese populations was mainly plant based diets, with over 80% of caloric intake coming from carbohydrates, and consumption of protein and fat was low [29]. In association with considerable improvement in the country's economic situation over the past two decades, there were marked improvements in the population's dietary intake. The average daily intake of animal products, especially meat, has increased considerably from 24 grams/day in 1990 to 51 grams/day in 2000. The percentage of energy from fat has risen from 6% in 1990 to 12% by 2002 and in many urban groups, the figure has exceeded 20% [24]. The previous RDA for Vietnam made no recommendation for intake of macronutrients by specific age groups. A general recommendation in the 2000 RDA on percentage of energy intake derived from protein, fat and carbohydrate for Vietnamese was 12%, 18%, and 70% respectively. For the 2006 RDA, there was an increase in the recommended fat intake for children from 4 to 5 years (18–25%, upper limit: 30%). Therefore, they are considered to be suitable for use in our study.

### Limitations of the study

There are a number of limitations of this study that should be considered when interpreting our findings. FFQs are designed to estimate an individual's usual intake of food, and are suitable for ranking subjects according to food or nutrient intake rather than for estimating the levels of intake [14]. Furthermore, validation studies of FFQs for measuring dietary habits of young children have shown that they overestimate the mean energy intake by 0.2% to 25% [30,31] and this could also be the case for this child population. However, our interpretation was based on the mean nutrient intakes, expressed as a percentage of energy from macronutrients, rather than the absolute levels of intake. Additionally, comparison of nutrient intake across different groups has relied on the ranking characteristic of dietary data collected from the FFQs.

Since this study was performed in the preschool setting with an attendance rate for children aged four to five years at about 80%, so there was another 20% of children who did not attend these childcare centres and thus were not available for recruitment for this study. In addition, only children from urban areas of HCMC were recruited. Therefore, generalization of the findings of this study should be limited to children enrolled in preschools in urban HCMC.

## Conclusion

The findings of this study indicate an imbalance in nutrient intake in this child population in HCMC. Protein and fat consumption, particularly animal protein and fat intake, was higher than recommended, and there were lower levels of carbohydrate consumption. Such imbalances in nutrient intake appear to be more evident among boys than girls. Further examination is required to identify the dietary patterns causing this nutrient imbalance, and to monitor changes in dietary patterns to provide evidence for developing healthy eating programs in the future, as a part of an obesity prevention program for young children in HCMC.

## Competing interests

The authors declare that they have no competing interests.

## Authors' contributions

All authors have made significant contributions to the study and the manuscript and all authors are in agreement with the content of the manuscript.

## Pre-publication history

The pre-publication history for this paper can be accessed here:



## Supplementary Material

Additional file 1 **Appendix.** Click here for file
