# Supplementary material for: Energy and macronutrient intakes in preschool children in urban areas of Ho Chi Minh City, Vietnam
Source: BMC Pediatr. 2008 Oct 18;8:44. doi: 10.1186/1471-2431-8-44 (PMC2575195; doi:10.1186/1471-2431-8-44)
Supplement: Additional file 1 — Appendix. [file 1471-2431-8-44-S1.doc]

## FOOD FREQUENCY QUESTIONNAIRE

| 1 | **a** | **Glutinous rice with jambon, pork pemmican, Chinese sausage.** | **b** | **On average how much does the child eat?** |
| --- | --- | --- | --- | --- |
|  | 0 [ ] | Never | 6 [ ] | 1 soup spoon (picture) |
|  | 1 [ ] | 1-2 times/month | 7 [ ] | 1/4 portion (picture) |
|  | 2 [ ] | 3-4 times/month | 8 [ ] | 1/2 portion (picture) |
|  | 3 [ ] | 1-2 times/week | 9 [ ] | 3/4 portion (picture) |
|  | 4 [ ] | 3-4 times/week | 10 [ ] | 1 portion (picture) |
|  | 5 [ ] | 5-6 times/week |  |  |
| 2 | **a** | **Glutinous rice with mungo bean, black bean, maize seeds, and sugar** | **b** | **On average how much does the child eat?** |
|  | 0 [ ] | Never | 6 [ ] | 1 soup spoon (picture) |
|  | 1 [ ] | 1-2 times/month | 7 [ ] | 1/4 portion (picture) |
|  | 2 [ ] | 3-4 times/month | 8 [ ] | 1/2 portion (picture) |
|  | 3 [ ] | 1-2 times/week | 9 [ ] | 3/4 portion (picture) |
|  | 4 [ ] | 3-4 times/week | 10 [ ] | 1 portion (picture) |
|  | 5 [ ] | 5-6 times/week |  |  |
| 3 | **a** | **Glutinous rice cake with mungo bean paste or banana and sugar** | **b** | **On average how much does the child eat?** |
|  | 0 [ ] | Never | 6 [ ] | 1 soup spoon (picture) |
|  | 1 [ ] | 1-2 times/month | 7 [ ] | 1/4 portion (picture) |
|  | 2 [ ] | 3-4 times/month | 8 [ ] | 1/2 portion (picture) |
|  | 3 [ ] | 1-2 times/week | 9 [ ] | 3/4 portion (picture) |
|  | 4 [ ] | 3-4 times/week | 10 [ ] | 1 portion (picture) |
|  | 5 [ ] | 5-6 times/week |  |  |
| 4 | **a** | **Glutinous rice cake with mungo bean paste and pork meat** | **b** | **On average how much does the child eat?** |
|  | 0 [ ] | Never | 6 [ ] | 1 soup spoon (picture) |
|  | 1 [ ] | 1-2 times/month | 7 [ ] | 1/4 portion (picture) |
|  | 2 [ ] | 3-4 times/month | 8 [ ] | 1/2 portion (picture) |
|  | 3 [ ] | 1-2 times/week | 9 [ ] | 3/4 portion (picture) |
|  | 4 [ ] | 3-4 times/week | 10 [ ] | 1 portion (picture) |
|  | 5 [ ] | 5-6 times/week |  |  |
| 5 | **a** | **Mixture of pork and vegetable rolled in rice pancake, steamed** | **b** | **On average how much does the child eat?** |
|  | 0 [ ] | Never | 6 [ ] | 1 soup spoon (picture) |
|  | 1 [ ] | 1-2 times/month | 7 [ ] | 1/4 portion (picture) |
|  | 2 [ ] | 3-4 times/month | 8 [ ] | 1/2 portion (picture) |
|  | 3 [ ] | 1-2 times/week | 9 [ ] | 3/4 portion (picture) |
|  | 4 [ ] | 3-4 times/week | 10 [ ] | 1 portion (picture) |
|  | 5 [ ] | 5-6 times/week |  |  |
| 6 | **a** | **Dumpling, filled with mixture pork and vegetable** | **b** | **On average how much does the child eat?** |
|  | 0 [ ] | Never | 6 [ ] | 1 soup spoon (picture) |
|  | 1 [ ] | 1-2 times/month | 7 [ ] | 1/4 portion (picture) |
|  | 2 [ ] | 3-4 times/month | 8 [ ] | 1/2 portion (picture) |
|  | 3 [ ] | 1-2 times/week | 9 [ ] | 3/4 portion (picture) |
|  | 4 [ ] | 3-4 times/week | 10 [ ] | 1 portion (picture) |
|  | 5 [ ] | 5-6 times/week |  |  |
| 7 | **a** | **Bread filled with pork meat, pork meat** **paste, and vegetable** | **b** | **On average how much does the child eat?** |
|  | 0 [ ] | Never | 6 [ ] | a piece |
|  | 1 [ ] | Once-twice/month | 7 [ ] | 1/4 loaf |
|  | 2 [ ] | 3-4 times/month | 8 [ ] | 1/2 loaf |
|  | 3 [ ] | Once-twice/week | 9 [ ] | 3/4 loaf |
|  | 4 [ ] | 3-4 times/week | 10 [ ] | 1 loaf |
|  | 5 [ ] | 5-6 times/week |  |  |
| 8 | **a** | **Pho: Vietnamese beef noodle soup** | **b** | **On average how much does the child eat?** |
|  | 0 [ ] | Never | 6 [ ] | 1/4 bowl 1 (picture) |
|  | 1 [ ] | 1-2 times/month | 7 [ ] | 1/2 bowl 1 (picture) |
|  | 2 [ ] | 3-4 times/month | 8 [ ] | 3/4 bowl 1 (picture) |
|  | 3 [ ] | 1-2 times/week | 9 [ ] | 1 bowl 1 (picture) |
|  | 4 [ ] | 3-4 times/week | 10 [ ] | 1 bowl 2 (picture) |
|  | 5 [ ] | 5-6 times/week |  |  |
| 9 | **a** | **Hu tiu: Vietnamese clear noodle soup with pork, shrimp or quail egg** | **b** | **On average how much does the child eat?** |
|  | 0 [ ] | Never | 6 [ ] | 1/4 bowl 1 (picture) |
|  | 1 [ ] | 1-2 times/month | 7 [ ] | 1/2 bowl 1 (picture) |
|  | 2 [ ] | 3-4 times/month | 8 [ ] | 3/4 bowl 1 (picture) |
|  | 3 [ ] | 1-2 times/week | 9 [ ] | 1 bowl 1 (picture) |
|  | 4 [ ] | 3-4 times/week | 10 [ ] | 1 bowl 2 (picture) |
|  | 5 [ ] | 5-6 times/week |  |  |
| 10 | **a** | **Bun rieu: tomato vermicelli soup with fried tofu, river crab paste and ground shrimp** | **b** | **On average how much does the child eat?** |
|  | 0 [ ] | Never | 6 [ ] | 1/4 bowl 1 (picture) |
|  | 1 [ ] | 1-2 times/month | 7 [ ] | 1/2 bowl 1 (picture) |
|  | 2 [ ] | 3-4 times/month | 8 [ ] | 3/4 bowl 1 (picture) |
|  | 3 [ ] | 1-2 times/week | 9 [ ] | 1 bowl 1 (picture) |
|  | 4 [ ] | 3-4 times/week | 10 [ ] | 1 bowl 2 (picture) |
|  | 5 [ ] | 5-6 times/week |  |  |
| 11 | **a** | **Wheat noodle soup with pork or shrimp** | **b** | **On average how much does the child eat?** |
|  | 0 [ ] | Never | 6 [ ] | 1/4 bowl 1 (picture) |
|  | 1 [ ] | 1-2 times/month | 7 [ ] | 1/2 bowl 1 (picture) |
|  | 2 [ ] | 3-4 times/month | 8 [ ] | 3/4 bowl 1 (picture) |
|  | 3 [ ] | 1-2 times/week | 9 [ ] | 1 bowl 1 (picture) |
|  | 4 [ ] | 3-4 times/week | 10 [ ] | 1 bowl 2 (picture) |
|  | 5 [ ] | 5-6 times/week k |  |  |
| 12 | **a** | **Sweet bean soup ( black, white, mungo bean)** | **b** | **On average how much does the child eat?** |
|  | 0 [ ] | Never | 6 [ ] | 1/4 bowl 1 (picture) |
|  | 1 [ ] | 1-2 times/month | 7 [ ] | 1/2 bowl 1 (picture) |
|  | 2 [ ] | 3-4 times/month | 8 [ ] | 3/4 bowl 1 (picture) |
|  | 3 [ ] | 1-2 times/week | 9 [ ] | 1 bowl 1 (picture) |
|  | 4 [ ] | 3-4 times/week | 10 [ ] | 1 bowl 2 (picture) |
|  | 5 [ ] | 5-6 times/week |  |  |
| 13 | **a** | **Sweet with sticky rice, white bean, coconut milk** | **b** | **On average how much does the child eat?** |
|  | 0 [ ] | Never | 6 [ ] | 1/4 bowl 1 (picture) |
|  | 1 [ ] | 1-2 times/month | 7 [ ] | 1/2 bowl 1 (picture) |
|  | 2 [ ] | 3-4 times/month | 8 [ ] | 3/4 bowl 1 (picture) |
|  | 3 [ ] | 1-2 times/week | 9 [ ] | 1 bowl 1 (picture) |
|  | 4 [ ] | 3-4 times/week | 10 [ ] | 1 bowl 2 (picture) |
|  | 5 [ ] | 5-6 times/week |  |  |
| 14 | **a** | **Sweet soup with green bean paste** | **b** | **On average how much does the child eat?** |
|  | 0 [ ] | Never | 6 [ ] | 1/4 bowl 1 (picture) |
|  | 1 [ ] | 1-2 times/month | 7 [ ] | 1/2 bowl 1 (picture) |
|  | 2 [ ] | 3-4 times/month | 8 [ ] | 3/4 bowl 1 (picture) |
|  | 3 [ ] | 1-2 times/week | 9 [ ] | 1 bowl 1 (picture) |
|  | 4 [ ] | 3-4 times/week | 10 [ ] | 1 bowl 2 (picture) |
|  | 5 [ ] | 5-6 times/week |  |  |
| 15 | **a** | **Steamed rice** | **b** | **On average how much does the child eat?** |
|  | 0 [ ] | Never | 6 [ ] | 1/4 bowl 1 (picture) |
|  | 1 [ ] | 1-2 times/month | 7 [ ] | 1/2 bowl 1 (picture) |
|  | 2 [ ] | 3-4 times/month | 8 [ ] | 3/4 bowl 1 (picture) |
|  | 3 [ ] | 1-2 times/week | 9 [ ] | 1 bowl 1 (picture) |
|  | 4 [ ] | 3-4 times/week | 10 [ ] | 2 bowl 1 (picture) |
|  | 5 [ ] | 5-6 times/week |  |  |
|  | 6 [ ] | Everyday |  |  |
| 16 | **a** | **Rice gruel** | **b** | **On average how much does the child eat?** |
|  | 0 [ ] | Never | 6 [ ] | 1/4 bowl 1 (picture) |
|  | 1 [ ] | 1-2 times/month | 7 [ ] | 1/2 bowl 1 (picture) |
|  | 2 [ ] | 3-4 times/month | 8 [ ] | 3/4 bowl 1 (picture) |
|  | 3 [ ] | 1-2 times/week | 9 [ ] | 1 bowl 1 (picture) |
|  | 4 [ ] | 3-4 times/week | 10 [ ] | 2 bowl 1 (picture) |
|  | 5 [ ] | 5-6 times/week |  |  |
| 17 | **a** | **Kinh Do bread** | **b** | **On average how much does the child eat?** |
|  | 0 [ ] | Never | 6 [ ] | 1/4 small portion (picture) |
|  | 1 [ ] | 1-2 times/month | 7 [ ] | 1/2 small portion (picture) |
|  | 2 [ ] | 3-4 times/month | 8 [ ] | 1 small portion (picture) |
|  | 3 [ ] | 1-2 times/week | 9 [ ] | 3/4 average portion (picture) |
|  | 4 [ ] | 3-4 times/week | 10 [ ] | 1 average portion (picture) |
|  | 5 [ ] | 5-6 times/week |  |  |
| 18 | **a** | **Maize (boiled, steamed, baked)** | **b** | **On average how much does the child eat?** |
|  | 0 [ ] | Never | 6 [ ] | 1 soup spoon (picture) |
|  | 1 [ ] | 1-2 times/month | 7 [ ] | 1/4 portion (picture) |
|  | 2 [ ] | 3-4 times/month | 8 [ ] | 1/2 portion (picture) |
|  | 3 [ ] | 1-2 times/week | 9 [ ] | 3/4 portion (picture) |
|  | 4 [ ] | 3-4 times/week | 10 [ ] | 1 portion (picture) |
|  | 5 [ ] | 5-6 times/week |  |  |
| 19 | **a** | **Sweet potato, Indian taro** | **b** | **On average how much does the child eat?** |
|  | 0 [ ] | Never | 6 [ ] | 1/4 portion (picture) |
|  | 1 [ ] | 1-2 times/month | 7 [ ] | 1/2 portion (picture) |
|  | 2 [ ] | 3-4 times/month | 8 [ ] | 1 portion (picture) |
|  | 3 [ ] | 1-2 times/week | 9 [ ] | 1.5 portion (picture) |
|  | 4 [ ] | 3-4 times/week | 10 [ ] | 2 portion (picture) |
|  | 5 [ ] | 5-6 times/week |  |  |
| 20 | **a** | **Cassava** | **b** | **On average how much does the child eat?** |
|  | 0 [ ] | Never | 6 [ ] | 1/4 portion (picture) |
|  | 1 [ ] | 1-2 times/month | 7 [ ] | 1/2 portion (picture) |
|  | 2 [ ] | 3-4 times/month | 8 [ ] | 1 portion (picture) |
|  | 3 [ ] | 1-2 times/week | 9 [ ] | 1.5 portion (picture) |
|  | 4 [ ] | 3-4 times/week | 10 [ ] | 2 portion (picture) |
|  | 5 [ ] | 5-6 times/week |  |  |
| 21 | **a** | **Potato** | **b** | **On average how much does the child eat?** |
|  | 0 [ ] | Never | 6 [ ] | 1/4 portion (picture) |
|  | 1 [ ] | 1-2 times/month | 7 [ ] | 1/2 portion (picture) |
|  | 2 [ ] | 3-4 times/month | 8 [ ] | 1 portion (picture) |
|  | 3 [ ] | 1-2 times/week | 9 [ ] | 1.5 portion (picture) |
|  | 4 [ ] | 3-4 times/week | 10 [ ] | 2 portion (picture) |
|  | 5 [ ] | 5-6 times/week |  |  |
| 22 | **a** | **Tofu, fresh** | **b** | **On average how much does the child eat?** |
|  | 0 [ ] | Never | 6 [ ] | 1 soupspoon (picture) |
|  | 1 [ ] | 1-2 times/month | 7 [ ] | 1/4 portion (picture) |
|  | 2 [ ] | 3-4 times/month | 8 [ ] | 1/2 portion (picture) |
|  | 3 [ ] | 1-2 times/week | 9 [ ] | 3/4 portion (picture) |
|  | 4 [ ] | 3-4 times/week | 10 [ ] | 1 portion (picture) |
|  | 5 [ ] | 5-6 times/week |  |  |
| 23 | **a** | **Tofu, fried** | **b** | **On average how much does the child eat?** |
|  | 0 [ ] | Never | 6 [ ] | 1 soupspoon (picture) |
|  | 1 [ ] | 1-2 times/month | 7 [ ] | 1/4 portion (picture) |
|  | 2 [ ] | 3-4 times/month | 8 [ ] | 1/2 portion (picture) |
|  | 3 [ ] | 1-2 times/week | 9 [ ] | 3/4 portion (picture) |
|  | 4 [ ] | 3-4 times/week | 10 [ ] | 1 portion (picture) |
|  | 5 [ ] | 5-6 times/week |  |  |
| 24 | **a** | **Tofu in syrup** | **b** | **On average how much does the child eat?** |
|  | 0 [ ] | Never | 6 [ ] | 1 soupspoon (picture) |
|  | 1 [ ] | 1-2 times/month | 7 [ ] | 1/4 bowl (picture) |
|  | 2 [ ] | 3-4 times/month | 8 [ ] | 1/2 bowl (picture) |
|  | 3 [ ] | 1-2 times/week | 9 [ ] | 3/4 bowl (picture) |
|  | 4 [ ] | 3-4 times/week | 10 [ ] | 1 bowl (picture) |
|  | 5 [ ] | 5-6 times/week |  |  |
| 25 | **a** | **Instant noodle** | **b** | **On average how much does the child eat?** |
|  | 0 [ ] | Never | 6 [ ] | 1 soup spoon (picture) |
|  | 1 [ ] | 1-2 times/month | 7 [ ] | 1/4 portion (picture) |
|  | 2 [ ] | 3-4 times/month | 8 [ ] | 1/2 portion (picture) |
|  | 3 [ ] | 1-2 times/week | 9 [ ] | 3/4 portion (picture) |
|  | 4 [ ] | 3-4 times/week | 10 [ ] | 1 portion (picture) |
|  | 5 [ ] | 5-6 times/week |  |  |
| 26 | **a** | **Beef meat** | **b** | **On average how much does the child eat?** |
|  | 0 [ ] | Never | 7 [ ] | 1/2 soupspoon (picture) |
|  | 1 [ ] | 1-2 times/month | 8 [ ] | 1 soupspoon (picture) |
|  | 2 [ ] | 3-4 times/month | 9 [ ] | 1.5 soupspoons (picture) |
|  | 3 [ ] | 1-2 times/week | 10 [ ] | 2 soupspoons (picture) |
|  | 4 [ ] | 3-4 times/week | 11 [ ] | 3 soupspoons (picture) |
|  | 5 [ ] | 5-6 times/week |  |  |
|  | 6 [ ] | Everyday |  |  |
| 27 | **a** | **Lean pork meat** | **b** | **On average how much does the child eat?** |
|  | 0 [ ] | Never | 7 [ ] | 1/2 soupspoon (picture) |
|  | 1 [ ] | 1-2 times/month | 8 [ ] | 1 soupspoon (picture) |
|  | 2 [ ] | 3-4 times/month | 9 [ ] | 1.5 soupspoons (picture) |
|  | 3 [ ] | 1-2 times/week | 10 [ ] | 2 soupspoons (picture) |
|  | 4 [ ] | 3-4 times/week | 11 [ ] | 3 soupspoons (picture) |
|  | 5 [ ] | 5-6 times/week |  |  |
|  | 6 [ ] | Everyday |  |  |
| 28 | **a** | **Fat and lean pork meat** | **b** | **On average how much does the child eat?** |
|  | 0 [ ] | Never | 6 [ ] | 1/2 soupspoon (picture) |
|  | 1 [ ] | 1-2 times/month | 7 [ ] | 1 soupspoon (picture) |
|  | 2 [ ] | 3-4 times/month | 8 [ ] | 1.5 soupspoons (picture) |
|  | 3 [ ] | 1-2 times/week | 9 [ ] | 2 soupspoons (picture) |
|  | 4 [ ] | 3-4 times/week | 10 [ ] | 3 soupspoons (picture) |
|  | 5 [ ] | 5-6 times/week |  |  |
| 29 | **a** | **Chicken (bone removed)** | **b** | **On average how much does the child eat?** |
|  | 0 [ ] | Never | 6 [ ] | 1/2 soupspoon (picture) |
|  | 1 [ ] | 1-2 times/month | 7 [ ] | 1 soupspoon (picture) |
|  | 2 [ ] | 3-4 times/month | 8 [ ] | 1.5 soupspoons (picture) |
|  | 3 [ ] | 1-2 times/week | 9 [ ] | 2 soupspoons (picture) |
|  | 4 [ ] | 3-4 times/week | 10 [ ] | 3 soupspoons (picture) |
|  | 5 [ ] | 5-6 times/week |  |  |
| 30 | **a** | **Duck meat (bone removed)** | **b** | **On average how much does the child eat?** |
|  | 0 [ ] | Never | 6 [ ] | 1/2 soupspoon (picture) |
|  | 1 [ ] | 1-2 times/month | 7 [ ] | 1 soupspoon (picture) |
|  | 2 [ ] | 3-4 times/month | 8 [ ] | 1.5 soupspoons (picture) |
|  | 3 [ ] | 1-2 times/week | 9 [ ] | 2 soupspoons (picture) |
|  | 4 [ ] | 3-4 times/week | 10 [ ] | 3 soupspoons (picture) |
|  | 5 [ ] | 5-6 times/week |  |  |
| 31 | **a** | **Quail** | **b** | **On average how much does the child eat?** |
|  | 0 [ ] | Never | 6 [ ] | 1 soupspoon (picture) |
|  | 1 [ ] | Once-twice/month | 7 [ ] | 1 drumstick (picture) |
|  | 2 [ ] | 3-4 times/month | 8 [ ] | 2 drumsticks (picture) |
|  | 3 [ ] | Once-twice/week | 9 [ ] | 1/2 portion (picture) |
|  | 4 [ ] | 3-4 times/week | 10 [ ] | 1 portion (picture) |
|  | 5 [ ] | 5-6 times/week |  |  |
| 32 | **a** | **Chicken, duck liver** | **b** | **On average how much does the child eat?** |
|  | 0 [ ] | Never | 6 [ ] | 1 soup spoon (picture) |
|  | 1 [ ] | Once-twice/month | 7 [ ] | 1/4 portion (picture) |
|  | 2 [ ] | 3-4 times/month | 8 [ ] | 1/2 portion (picture) |
|  | 3 [ ] | Once-twice/week | 9 [ ] | 3/4 portion (picture) |
|  | 4 [ ] | 3-4 times/week | 10 [ ] | 1 portion (picture) |
|  | 5 [ ] | 5-6 times/week |  |  |
| 33 | **a** | **Hog liver** | **b** | **On average how much does the child eat?** |
|  | 0 [ ] | Never | 6 [ ] | 1 soupspoon (picture) |
|  | 1 [ ] | 1-2 times/month | 7 [ ] | 2 soupspoons (picture) |
|  | 2 [ ] | 3-4 times/month | 8 [ ] | 1 slice (picture) |
|  | 3 [ ] | 1-2 times/week | 9 [ ] | 2 slices (picture) |
|  | 4 [ ] | 3-4 times/week | 10 [ ] | 3 slices (picture) |
|  | 5 [ ] | 5-6 times/week |  |  |
| 34 | **a** | **Hog brain** | **b** | **On average how much does the child eat?** |
|  | 0 [ ] | Never | 6 [ ] | 1 soupspoon (picture) |
|  | 1 [ ] | 1-2 times/month | 7 [ ] | 2 soupspoons (picture) |
|  | 2 [ ] | 3-4 times/month | 8 [ ] | 3 soupspoons (picture) |
|  | 3 [ ] | 1-2 times/week | 9 [ ] | 1/4 bowl (picture) |
|  | 4 [ ] | 3-4 times/week | 10 [ ] | 1/2 bowl (picture) |
|  | 5 [ ] | 5-6 times/week |  |  |
| 35 | **a** | **Chicken heart** | **b** | **On average how much does the child eat?** |
|  | 0 [ ] | Never | 6 [ ] | 1 soupspoon (picture) |
|  | 1 [ ] | 1-2 times/month | 7 [ ] | 2 soupspoons (picture) |
|  | 2 [ ] | 3-4 times/month | 8 [ ] | 1 slice (picture) |
|  | 3 [ ] | 1-2 times/week | 9 [ ] | 2 slices (picture) |
|  | 4 [ ] | 3-4 times/week | 10 [ ] | 3 slices (picture) |
|  | 5 [ ] | 5-6 times/week |  |  |
| 36 | **a** | **Hog heart** | **b** | **On average how much does the child eat?** |
|  | 0 [ ] | Never | 6 [ ] | 1 soupspoon (picture) |
|  | 1 [ ] | 1-2 times/month | 7 [ ] | 1/4 portion (picture) |
|  | 2 [ ] | 3-4 times/month | 8 [ ] | 1/2 portion (picture) |
|  | 3 [ ] | 1-2 times/week | 9 [ ] | 3/4 portion (picture) |
|  | 4 [ ] | 3-4 times/week | 10 [ ] | 1 portion (picture) |
|  | 5 [ ] | 5-6 times/week k |  |  |
| 37 | **a** | **Lean pork paste, steamed** | **b** | **On average how much does the child eat?** |
|  | 0 [ ] | Never | 6 [ ] | 1 soupspoon (picture) |
|  | 1 [ ] | 1-2 times/month | 7 [ ] | 1 slice (picture) |
|  | 2 [ ] | 3-4 times/month | 8 [ ] | 2 slices (picture) |
|  | 3 [ ] | 1-2 times/week | 9 [ ] | 3 slices (picture) |
|  | 4 [ ] | 3-4 times/week | 10 [ ] | 1 section |
|  | 5 [ ] | 5-6 times/week |  |  |
| 38 | **a** | **Sausage** | **b** | **On average how much does the child eat?** |
|  | 0 [ ] | Never | 6 [ ] | 1/4 portion (picture) |
|  | 1 [ ] | 1-2 times/month | 7 [ ] | 1/2 portion (picture) |
|  | 2 [ ] | 3-4 times/month | 8 [ ] | 3/4 portion (picture) |
|  | 3 [ ] | 1-2 times/week | 9 [ ] | 1 portion (picture) |
|  | 4 [ ] | 3-4 times/week | 10 [ ] | 2 portion (picture) |
|  | 5 [ ] | 5-6 times/week |  |  |
| 39 | **a** | **Pork pemmican** | **b** | **On average how much does the child eat?** |
|  | 0 [ ] | Never | 6 [ ] | 1/2 soupspoon (picture) |
|  | 1 [ ] | 1-2 times/month | 7 [ ] | 1 soupspoon (picture) |
|  | 2 [ ] | 3-4 times/month | 8 [ ] | 1.5 soupspoons (picture) |
|  | 3 [ ] | 1-2 times/week | 9 [ ] | 2 soupspoons (picture) |
|  | 4 [ ] | 3-4 times/week | 10 [ ] | 3 soupspoons (picture) |
|  | 5 [ ] | 5-6 times/week |  |  |
| 40 | **a** | **Whole milk, fresh no sugar** | **b** | **On average how much does the child eat?** |
|  | 0 [ ] | Never | 6 [ ] | 2 spoons (60 ml) (picture) |
|  | 1 [ ] | 1-2 times/month | 7 [ ] | 3 spoons (120 ml) (picture) |
|  | 2 [ ] | 3-4 times/month | 8 [ ] | 1/2 cup (picture) |
|  | 3 [ ] | 1-2 times/week | 9 [ ] | 1/2 portion (picture) |
|  | 4 [ ] | 3-4 times/week | 10 [ ] | 1 portion (picture) |
|  | 5 [ ] | 5-6 times/week |  |  |
| 41 | **a** | **Whole milk, fresh with sugar** | **b** | **On average how much does the child eat?** |
|  | 0 [ ] | Never | 6 [ ] | 2 spoons (60 ml) (picture) |
|  | 1 [ ] | 1-2 times/month | 7 [ ] | 3 spoons (120 ml) (picture) |
|  | 2 [ ] | 3-4 times/month | 8 [ ] | 1/2 cup (picture) |
|  | 3 [ ] | 1-2 times/week | 9 [ ] | 1/2 portion (picture) |
|  | 4 [ ] | 3-4 times/week | 10 [ ] | 1 portion (picture) |
|  | 5 [ ] | 5-6 times/week |  |  |
| 42 | **a** | **Full cream milk, powder** | **b** | **On average how much does the child eat?** |
|  | 0 [ ] | Never | 6 [ ] | 2 spoons (60 ml) (picture) |
|  | 1 [ ] | 1-2 times/month | 7 [ ] | 3 spoons (120 ml) (picture) |
|  | 2 [ ] | 3-4 times/month | 8 [ ] | 1/2 cup (picture) |
|  | 3 [ ] | 1-2 times/week | 9 [ ] | 3/4 cup (picture) |
|  | 4 [ ] | 3-4 times/week | 10 [ ] | 1 cup (picture) |
|  | 5 [ ] | 5-6 times/week |  |  |
| 43 | **a** | **Skim milk, powder** | **b** | **On average how much does the child eat?** |
|  | 0 [ ] | Never | 6 [ ] | 2 spoons (60 ml) (picture) |
|  | 1 [ ] | 1-2 times/month | 7 [ ] | 3 spoons (120 ml) (picture) |
|  | 2 [ ] | 3-4 times/month | 8 [ ] | 1/2 cup (picture) |
|  | 3 [ ] | 1-2 times/week | 9 [ ] | 3/4 cup (picture) |
|  | 4 [ ] | 3-4 times/week | 10 [ ] | 1 cup (picture) |
|  | 5 [ ] | 5-6 times/week |  |  |
| 44 | **a** | **Yoghurt** | **b** | **On average how much does the child eat?** |
|  | 0 [ ] | Never | 6 [ ] | 1 soupspoon |
|  | 1 [ ] | 1-2 times/month | 7 [ ] | 1/2 portion (picture) |
|  | 2 [ ] | 3-4 times/month | 8 [ ] | 1 portion (picture) |
|  | 3 [ ] | 1-2 times/week | 9 [ ] | 1.5 portion (picture) |
|  | 4 [ ] | 3-4 times/week | 10 [ ] | 2 portions (picture) |
|  | 5 [ ] | 5-6 times/week |  |  |
| 45 | **a** | **Condensed milk, sweetened** | **b** | **On average how much does the child eat?** |
|  | 0 [ ] | Never | 6 [ ] | 1 teaspoon (picture) |
|  | 1 [ ] | 1-2 times/month | 7 [ ] | 2 teaspoons (picture) |
|  | 2 [ ] | 3-4 times/month | 8 [ ] | 3 teaspoons (picture) |
|  | 3 [ ] | 1-2 times/week | 9 [ ] | 1/2 cup (picture) |
|  | 4 [ ] | 3-4 times/week | 10 [ ] | 1 cup (picture) |
|  | 5 [ ] | 5-6 times/week |  |  |
| 46 | **a** | **Soy milk** | **b** | **On average how much does the child eat?** |
|  | 0 [ ] | Never | 6 [ ] | 1 small cup (picture) |
|  | 1 [ ] | 1-2 times/month | 7 [ ] | 1/2 cup (picture) |
|  | 2 [ ] | 3-4 times/month | 8 [ ] | 1/4 portion (picture) |
|  | 3 [ ] | 1-2 times/week | 9 [ ] | 1/2 portion (picture) |
|  | 4 [ ] | 3-4 times/week | 10 [ ] | 1 portion (picture) |
|  | 5 [ ] | 5-6 times/week |  |  |
| 47 | **a** | **Chicken, duck egg** | **b** | **On average how much does the child eat?** |
|  | 0 [ ] | Never | 6 [ ] | 1/4 egg (picture) |
|  | 1 [ ] | 1-2 times/month | 7 [ ] | 1/2 egg (picture) |
|  | 2 [ ] | 3-4 times/month | 8 [ ] | 3/4 egg (picture) |
|  | 3 [ ] | 1-2 times/week | 9 [ ] | 1 egg (picture) |
|  | 4 [ ] | 3-4 times/week | 10 [ ] | 2 eggs (picture) |
|  | 5 [ ] | 5-6 times/week |  |  |
| 48 | **a** | **Half hatched duck-egg** | **b** | **On average how much does the child eat?** |
|  | 0 [ ] | Never | 6 [ ] | 1/4 egg (picture) |
|  | 1 [ ] | 1-2 times/month | 7 [ ] | 1/2 egg (picture) |
|  | 2 [ ] | 3-4 times/month | 8 [ ] | 3/4 egg (picture) |
|  | 3 [ ] | 1-2 times/week | 9 [ ] | 1 egg (picture) |
|  | 4 [ ] | 3-4 times/week | 10 [ ] | 2 eggs (picture) |
|  | 5 [ ] | 5-6 times/week |  |  |
| 49 | **a** | **Quail egg** | **b** | **On average how much does the child eat?** |
|  | 0 [ ] | Never | 6 [ ] | 1/2 egg (picture) |
|  | 1 [ ] | 1-2 times/month | 7 [ ] | 1 egg (picture) |
|  | 2 [ ] | 3-4 times/month | 8 [ ] | 2-3 eggs (picture) |
|  | 3 [ ] | 1-2 times/week | 9 [ ] | 4-5 eggs (picture) |
|  | 4 [ ] | 3-4 times/week | 10 [ ] | 6-7 eggs (picture) |
|  | 5 [ ] | 5-6 times/week |  |  |
| 50 | **a** | **Frog meat** | **b** | **On average how much does the child eat?** |
|  | 0 [ ] | Never | 6 [ ] | 1 soupspoon (picture) |
|  | 1 [ ] | 1-2 times/month | 7 [ ] | 1 leg (picture) |
|  | 2 [ ] | 3-4 times/month | 8 [ ] | 2-3 legs (picture) |
|  | 3 [ ] | 1-2 times/week | 9 [ ] | 4-5 legs (picture) |
|  | 4 [ ] | 3-4 times/week | 10 [ ] | 6-7 legs (picture) |
|  | 5 [ ] | 5-6 times/week |  |  |
| 51 | **a** | **Eel meat** | **b** | **On average how much does the child eat?** |
|  | 0 [ ] | Never | 6 [ ] | 1 soupspoon (picture) |
|  | 1 [ ] | 1-2 times/month | 7 [ ] | 1 section (picture) |
|  | 2 [ ] | 3-4 times/month | 8 [ ] | 2 sections (picture) |
|  | 3 [ ] | 1-2 times/week | 9 [ ] | 3 sections (picture) |
|  | 4 [ ] | 3-4 times/week | 10 [ ] | 4 sections (picture) |
|  | 5 [ ] | 5-6 times/week |  |  |
| 52 | **a** | **Fatty fish (bong lau, basa, tra fish)** | **b** | **On average how much does the child eat?** |
|  | 0 [ ] | Never | 6 [ ] | 1 soupspoon (picture) |
|  | 1 [ ] | 1-2 times/month | 7 [ ] | 1/4 slice (picture) |
|  | 2 [ ] | 3-4 times/month | 8 [ ] | 1/2 slice (picture) |
|  | 3 [ ] | 1-2 times/week | 9 [ ] | 1 slice (picture) |
|  | 4 [ ] | 3-4 times/week | 10 [ ] | 2 slices (picture) |
|  | 5 [ ] | 5-6 times/week |  |  |
| 53 | **a** | **Fat free fish (snack-head, mackerel, anabas)** | **b** | **On average how much does the child eat?** |
|  | 0 [ ] | Never | 6 [ ] | 1 soupspoon (picture) |
|  | 1 [ ] | 1-2 times/month | 7 [ ] | 1/4 slice (picture) |
|  | 2 [ ] | 3-4 times/month | 8 [ ] | 1/2 slice (picture) |
|  | 3 [ ] | 1-2 times/week | 9 [ ] | 1 slice (picture) |
|  | 4 [ ] | 3-4 times/week | 10 [ ] | 2 slices (picture) |
|  | 5 [ ] | 5-6 times/week |  |  |
| 54 | **a** | **Clam, oyster, helix** | **b** | **On average how much does the child eat?** |
|  | 0 [ ] | Never | 6 [ ] | 1-2 clams (picture) |
|  | 1 [ ] | 1-2 times/month | 7 [ ] | 3-4 clams (picture) |
|  | 2 [ ] | 3-4 times/month | 8 [ ] | 5-6 clams (picture) |
|  | 3 [ ] | 1-2 times/week | 9 [ ] | 7-8 clams (picture) |
|  | 4 [ ] | 3-4 times/week | 10 [ ] | 9-10 clams (picture) |
|  | 5 [ ] | 5-6 times/week |  |  |
| 55 | **a** | **Prawn** | **b** | **On average how much does the child eat?** |
|  | 0 [ ] | Never | 6 [ ] | 1 soupspoon (picture) |
|  | 1 [ ] | 1-2 times/month | 7 [ ] | 1 prawn (picture) |
|  | 2 [ ] | 3-4 times/month | 8 [ ] | 2 prawns (picture) |
|  | 3 [ ] | 1-2 times/week | 9 [ ] | 3 prawns (picture) |
|  | 4 [ ] | 3-4 times/week | 10 [ ] | 4 prawns (picture) |
|  | 5 [ ] | 5-6 times/week |  |  |
| 56 | **a** | **River crab** | **b** | **On average how much does the child eat?** |
|  | 0 [ ] | Never | 6 [ ] | 1/4 portion (picture) |
|  | 1 [ ] | 1-2 times/month | 7 [ ] | 1/2 portion (picture) |
|  | 2 [ ] | 3-4 times/month | 8 [ ] | 3/4 portion (picture) |
|  | 3 [ ] | 1-2 times/week | 9 [ ] | 1 portion (picture) |
|  | 4 [ ] | 3-4 times/week | 10 [ ] | 2 portion (picture) |
|  | 5 [ ] | 5-6 times/week |  |  |
| 57 | **a** | **Sea crab** | **b** | **On average how much does the child eat?** |
|  | 0 [ ] | Never | 6 [ ] | 1/4 portion (picture) |
|  | 1 [ ] | 1-2 times/month | 7 [ ] | 1/2 portion (picture) |
|  | 2 [ ] | 3-4 times/month | 8 [ ] | 3/4 portion (picture) |
|  | 3 [ ] | 1-2 times/week | 9 [ ] | 1 portion (picture) |
|  | 4 [ ] | 3-4 times/week | 10 [ ] | 2 portion (picture) |
|  | 5 [ ] | 5-6 times/week |  |  |
| 58 | **a** | **Calabash gourd, wax gourd** | **b** | **On average how much does the child eat?** |
|  | 0 [ ] | Never | 6 [ ] | 1 soupspoon (picture) |
|  | 1 [ ] | 1-2 times/month | 7 [ ] | 1/2 section (picture) |
|  | 2 [ ] | 3-4 times/month | 8 [ ] | 1 section (picture) |
|  | 3 [ ] | 1-2 times/week | 9 [ ] | 2 sections (picture) |
|  | 4 [ ] | 3-4 times/week | 10 [ ] | 3 sections (picture) |
|  | 5 [ ] | 5-6 times/week |  |  |
| 59 | **a** | **Pumpkin squash** | **b** | **On average how much does the child eat?** |
|  | 0 [ ] | Never | 6 [ ] | 1 soupspoon (picture) |
|  | 1 [ ] | 1-2 times/month | 7 [ ] | 1/2 section (picture) |
|  | 2 [ ] | 3-4 times/month | 8 [ ] | 1 section (picture) |
|  | 3 [ ] | 1-2 times/week | 9 [ ] | 2 sections (picture) |
|  | 4 [ ] | 3-4 times/week | 10 [ ] | 4 sections (picture) |
|  | 5 [ ] | 5-6 times/week |  |  |
| 60 | **a** | **Carrot** | **b** | **On average how much does the child eat?** |
|  | 0 [ ] | Never | 6 [ ] | 1 soupspoon (picture) |
|  | 1 [ ] | 1-2 times/month | 7 [ ] | 1/2 portion (picture) |
|  | 2 [ ] | 3-4 times/month | 8 [ ] | 1 (picture) |
|  | 3 [ ] | 1-2 times/week | 9 [ ] | 1.5 portions (picture) |
|  | 4 [ ] | 3-4 times/week | 10 [ ] | 2 portions (picture) |
|  | 5 [ ] | 5-6 times/week |  |  |
| 61 | **a** | **Cabbage** | **b** | **On average how much does the child eat?** |
|  | 0 [ ] | Never | 6 [ ] | 1 soupspoon (picture) |
|  | 1 [ ] | 1-2 times/month | 7 [ ] | 2 soupspoons (picture) |
|  | 2 [ ] | 3-4 times/month | 8 [ ] | 3 soupspoons (picture) |
|  | 3 [ ] | 1-2 times/week | 9 [ ] | 1/2 bowl (picture) |
|  | 4 [ ] | 3-4 times/week | 10 [ ] | 1 bowl (picture) |
|  | 5 [ ] | 5-6 times/week |  |  |
| 62 | **a** | **Yam bean** | **b** | **On average how much does the child eat?** |
|  | 0 [ ] | Never | 6 [ ] | 1 soupspoon (picture) |
|  | 1 [ ] | 1-2 times/month | 7 [ ] | 2 soupspoons (picture) |
|  | 2 [ ] | 3-4 times/month | 8 [ ] | 1/4 bowl (picture) |
|  | 3 [ ] | 1-2 times/week | 9 [ ] | 1/2 bowl (picture) |
|  | 4 [ ] | 3-4 times/week | 10 [ ] | 3/4 bowl (picture) |
|  | 5 [ ] | 5-6 times/week |  |  |
| 63 | **a** | **French bean** | **b** | **On average how much does the child eat?** |
|  | 0 [ ] | Never | 6 [ ] | 1 soupspoon (picture) |
|  | 1 [ ] | 1-2 times/month | 7 [ ] | 2 soupspoons (picture) |
|  | 2 [ ] | 3-4 times/month | 8 [ ] | 1/4 bowl (picture) |
|  | 3 [ ] | 1-2 times/week | 9 [ ] | 1/2 bowl (picture) |
|  | 4 [ ] | 3-4 times/week | 10 [ ] | 3/4 bowl (picture) |
|  | 5 [ ] | 5-6 times/week |  |  |
| 64 | **a** | **Mungo bean sprouts** | **b** | **On average how much does the child eat?** |
|  | 0 [ ] | Never | 6 [ ] | 1 soupspoon (picture) |
|  | 1 [ ] | 1-2 times/month | 7 [ ] | 1/4 bowl (picture) |
|  | 2 [ ] | 3-4 times/month | 8 [ ] | 1/2 bowl (picture) |
|  | 3 [ ] | 1-2 times/week | 9 [ ] | 3/4 bowl (picture) |
|  | 4 [ ] | 3-4 times/week | 10 [ ] | 1 bowl |
|  | 5 [ ] | 5-6 times/week |  |  |
| 65 | **a** | **Loofah** | **b** | **On average how much does the child eat?** |
|  | 0 [ ] | Never | 6 [ ] | 1 soupspoon (picture) |
|  | 1 [ ] | 1-2 times/month | 7 [ ] | 1/4 bowl (picture) |
|  | 2 [ ] | 3-4 times/month | 8 [ ] | 1/2 bowl (picture) |
|  | 3 [ ] | 1-2 times/week | 9 [ ] | 3/4 bowl (picture) |
|  | 4 [ ] | 3-4 times/week | 10 [ ] | 1 bowl |
|  | 5 [ ] | 5-6 times/week |  |  |
| 66 | **a** | **Green leave vegetable** | **b** | **On average how much does the child eat?** |
|  | 0 [ ] | Never | 6 [ ] | 1 soupspoon (picture) |
|  | 1 [ ] | 1-2 times/month | 7 [ ] | 1/4 bowl (picture) |
|  | 2 [ ] | 3-4 times/month | 8 [ ] | 1/2 bowl (picture) |
|  | 3 [ ] | 1-2 times/week | 9 [ ] | 3/4 bowl (picture) |
|  | 4 [ ] | 3-4 times/week | 10 [ ] | 1 bowl |
|  | 5 [ ] | 5-6 times/week |  |  |
| 67 | **a** | **Cauliflower** | **b** | **On average how much does the child eat?** |
|  | 0 [ ] | Never | 6 [ ] | 1 soupspoon (picture) |
|  | 1 [ ] | 1-2 times/month | 7 [ ] | 2 soupspoons (picture) |
|  | 2 [ ] | 3-4 times/month | 8 [ ] | 1/4 bowl (picture) |
|  | 3 [ ] | 1-2 times/week | 9 [ ] | 1/2 bowl (picture) |
|  | 4 [ ] | 3-4 times/week | 10 [ ] | 3/4 bowl (picture) |
|  | 5 [ ] | 5-6 times/week |  |  |
| 68 | **a** | **Mushroom** | **b** | **On average how much does the child eat?** |
|  | 0 [ ] | Never | 6 [ ] | 1 soupspoon (picture) |
|  | 1 [ ] | 1-2 times/month | 7 [ ] | 1/4 disk (picture) |
|  | 2 [ ] | 3-4 times/month | 8 [ ] | 1/2 disk (picture) |
|  | 3 [ ] | 1-2 times/week | 9 [ ] | 3/4 disk (picture) |
|  | 4 [ ] | 3-4 times/week | 10 [ ] | 1 disk (picture) |
|  | 5 [ ] | 5-6 times/week |  |  |
| 69 | **a** | **Grapefruit** | **b** | **On average how much does the child eat?** |
|  | 0 [ ] | Never | 6 [ ] | 1/4 portion (picture) |
|  | 1 [ ] | 1-2 times/month | 7 [ ] | 1/2 portion (picture) |
|  | 2 [ ] | 3-4 times/month | 8 [ ] | 1 portion (picture) |
|  | 3 [ ] | 1-2 times/week | 9 [ ] | 1.5 portions (picture) |
|  | 4 [ ] | 3-4 times/week | 10 [ ] | 2 portions (picture) |
|  | 5 [ ] | 5-6 times/week |  |  |
| 70 | **a** | **Orange** | **b** | **On average how much does the child eat?** |
|  | 0 [ ] | Never | 6 [ ] | 1 section (picture) |
|  | 1 [ ] | 1-2 times/month | 7 [ ] | 2 sections (picture) |
|  | 2 [ ] | 3-4 times/month | 8 [ ] | 3 sections (picture) |
|  | 3 [ ] | 1-2 times/week | 9 [ ] | 1/2 portion (picture) |
|  | 4 [ ] | 3-4 times/week | 10 [ ] | 1 portion (picture) |
|  | 5 [ ] | 5-6 times/week |  |  |
| 71 | **a** | **Mandarin** | **b** | **On average how much does the child eat?** |
|  | 0 [ ] | Never | 6 [ ] | 1 mandarin (picture) |
|  | 1 [ ] | 1-2 times/month | 7 [ ] | 2-3 mandarins (picture) |
|  | 2 [ ] | 3-4 times/month | 8 [ ] | 3 sections (picture) |
|  | 3 [ ] | 1-2 times/week | 9 [ ] | 1/2 portion (picture) |
|  | 4 [ ] | 3-4 times/week | 10 [ ] | 1 portion (picture) |
|  | 5 [ ] | 5-6 times/week |  |  |
| 72 | **a** | **Rambutan** | **b** | **On average how much does the child eat?** |
|  | 0 [ ] | Never | 6 [ ] | 1 rambutan (picture) |
|  | 1 [ ] | 1-2 times/month | 7 [ ] | 2-3 rambutans(picture) |
|  | 2 [ ] | 3-4 times/month | 8 [ ] | 4-5 rambutans (picture) |
|  | 3 [ ] | 1-2 times/week | 9 [ ] | 6-7 rambutants (picture) |
|  | 4 [ ] | 3-4 times/week | 10 [ ] | 8-10 rambutans (picture) |
|  | 5 [ ] | 5-6 times/week |  |  |
| 73 | **a** | **Banana** | **b** | **On average how much does the child eat?** |
|  | 0 [ ] | Never | 6 [ ] | 1 soupspoon (picture) |
|  | 1 [ ] | 1-2 times/month | 7 [ ] | 1/4 banana (picture) |
|  | 2 [ ] | 3-4 times/month | 8 [ ] | 1/2 banana (picture) |
|  | 3 [ ] | 1-2 times/week | 9 [ ] | 1 banana (picture) |
|  | 4 [ ] | 3-4 times/week | 10 [ ] | 2 bananas |
|  | 5 [ ] | 5-6 times/week |  |  |
| 74 | **a** | **Pine apple** | **b** | **On average how much does the child eat?** |
|  | 0 [ ] | Never | 6 [ ] | 1/4 portion (picture) |
|  | 1 [ ] | 1-2 times/month | 7 [ ] | 1/2 portion (picture) |
|  | 2 [ ] | 3-4 times/month | 8 [ ] | 3/4 portion (picture) |
|  | 3 [ ] | 1-2 times/week | 9 [ ] | 1 portion (picture) |
|  | 4 [ ] | 3-4 times/week | 10 [ ] | 1.5 portions (picture) |
|  | 5 [ ] | 5-6 times/week |  |  |
| 75 | **a** | **Papaya, ripen** | **b** | **On average how much does the child eat?** |
|  | 0 [ ] | Never | 6 [ ] | 1 soupspoon (picture) |
|  | 1 [ ] | 1-2 times/month | 7 [ ] | 1/4 portion (picture) |
|  | 2 [ ] | 3-4 times/month | 8 [ ] | 1/2 portion (picture) |
|  | 3 [ ] | 1-2 times/week | 9 [ ] | 3/4 portion (picture) |
|  | 4 [ ] | 3-4 times/week | 10 [ ] | 1 portion (picture) |
|  | 5 [ ] | 5-6 times/week |  |  |
| 76 | **a** | **Plum** | **b** | **On average how much does the child eat?** |
|  | 0 [ ] | Never | 6 [ ] | 1/4 plum (picture) |
|  | 1 [ ] | 1-2 times/month | 7 [ ] | 1/2 plum (picture) |
|  | 2 [ ] | 3-4 times/month | 8 [ ] | 1 plum (picture) |
|  | 3 [ ] | 1-2 times/week | 9 [ ] | 2-3 plums (picture) |
|  | 4 [ ] | 3-4 times/week | 10 [ ] | 4-5 plums (picture) |
|  | 5 [ ] | 5-6 times/week |  |  |
| 77 | **a** | **Pear** | **b** | **On average how much does the child eat?** |
|  | 0 [ ] | Never | 6 [ ] | 1 soupspoon (picture) |
|  | 1 [ ] | 1-2 times/month | 7 [ ] | 1/4 portion (picture) |
|  | 2 [ ] | 3-4 times/month | 8 [ ] | 1/2 portion (picture) |
|  | 3 [ ] | 1-2 times/week | 9 [ ] | 3/4 portion (picture) |
|  | 4 [ ] | 3-4 times/week | 10 [ ] | 1 portion (picture) |
|  | 5 [ ] | 5-6 times/week |  |  |
| 78 | **a** | **Jackfruit** | **b** | **On average how much does the child eat?** |
|  | 0 [ ] | Never | 6 [ ] | 1 soupspoon (picture) |
|  | 1 [ ] | 1-2 times/month | 7 [ ] | 1 section (picture) |
|  | 2 [ ] | 3-4 times/month | 8 [ ] | 2-3 sections (picture) |
|  | 3 [ ] | 1-2 times/week | 9 [ ] | 4-5 sections (picture) |
|  | 4 [ ] | 3-4 times/week | 10 [ ] | 6-7 sections (picture) |
|  | 5 [ ] | 5-6 times/week |  |  |
| 79 | **a** | **Mangosteen** | **b** | **On average how much does the child eat?** |
|  | 0 [ ] | Never | 6 [ ] | 1 soupspoon (picture) |
|  | 1 [ ] | 1-2 times/month | 7 [ ] | 1/2 mangosteen (picture) |
|  | 2 [ ] | 3-4 times/month | 8 [ ] | 1 mangosteen (picture) |
|  | 3 [ ] | 1-2 times/week | 9 [ ] | 2-3 mangosteens (picture) |
|  | 4 [ ] | 3-4 times/week | 10 [ ] | 4-5 mangosteens (picture) |
|  | 5 [ ] | 5-6 times/week |  |  |
| 80 | **a** | **Red persimmon, soft type** | **b** | **On average how much does the child eat?** |
|  | 0 [ ] | Never | 6 [ ] | 1 soupspoon (picture) |
|  | 1 [ ] | 1-2 times/month | 7 [ ] | 1/2 red persimmon (picture) |
|  | 2 [ ] | 3-4 times/month | 8 [ ] | 1 red persimmon (picture) |
|  | 3 [ ] | 1-2 times/week | 9 [ ] | 2-3 red persimmon(picture) |
|  | 4 [ ] | 3-4 times/week | 10 [ ] | 4-5 red persimmon (picture) |
|  | 5 [ ] | 5-6 times/week |  |  |
| 81 | **a** | **Longan fruit** | **b** | **On average how much does the child eat?** |
|  | 0 [ ] | Never | 6 [ ] | 2 longans (picture) |
|  | 1 [ ] | 1-2 times/month | 7 [ ] | 3-4 longans (picture) |
|  | 2 [ ] | 3-4 times/month | 8 [ ] | 5-6 longans (picture) |
|  | 3 [ ] | 1-2 times/week | 9 [ ] | 7-8 longans (picture) |
|  | 4 [ ] | 3-4 times/week | 10 [ ] | 9-10 longans (picture) |
|  | 5 [ ] | 5-6 times/week |  |  |
| 82 | **a** | **Avocado** | **b** | **On average how much does the child eat?** |
|  | 0 [ ] | Never | 6 [ ] | 1 soupspoon (picture) |
|  | 1 [ ] | 1-2 times/month | 7 [ ] | 1/4 portion (picture) |
|  | 2 [ ] | 3-4 times/month | 8 [ ] | 1/2 portion (picture) |
|  | 3 [ ] | 1-2 times/week | 9 [ ] | 3/4 portion (picture) |
|  | 4 [ ] | 3-4 times/week | 10 [ ] | 1 portion (picture) |
|  | 5 [ ] | 5-6 times/week |  |  |
| 83 | **a** | **Dragon’s fruit** | **b** | **On average how much does the child eat?** |
|  | 0 [ ] | Never | 6 [ ] | 1 soupspoon (picture) |
|  | 1 [ ] | 1-2 times/month | 7 [ ] | 1/2 portion (picture) |
|  | 2 [ ] | 3-4 times/month | 8 [ ] | 1 portion (picture) |
|  | 3 [ ] | 1-2 times/week | 9 [ ] | 1.5 portion (picture) |
|  | 4 [ ] | 3-4 times/week | 10 [ ] | 2 portion (picture) |
|  | 5 [ ] | 5-6 times/week |  |  |
| 84 | **a** | **Apple** | **b** | **On average how much does the child eat?** |
|  | 0 [ ] | Never | 6 [ ] | 1 soupspoon (picture) |
|  | 1 [ ] | 1-2 times/month | 7 [ ] | 1/4 portion (picture) |
|  | 2 [ ] | 3-4 times/month | 8 [ ] | 1/2 portion (picture) |
|  | 3 [ ] | 1-2 times/week | 9 [ ] | 3/4 portion (picture) |
|  | 4 [ ] | 3-4 times/week | 10 [ ] | 1 portion (picture) |
|  | 5 [ ] | 5-6 times/week |  |  |
| 85 | **a** | **Mango, ripen** | **b** | **On average how much does the child eat?** |
|  | 0 [ ] | Never | 6 [ ] | 1 soupspoon (picture) |
|  | 1 [ ] | 1-2 times/month | 7 [ ] | 1/2 section (picture) |
|  | 2 [ ] | 3-4 times/month | 8 [ ] | 1 section (picture) |
|  | 3 [ ] | 1-2 times/week | 9 [ ] | 1/2 mango (picture) |
|  | 4 [ ] | 3-4 times/week | 10 [ ] | 1 mango (picture) |
|  | 5 [ ] | 5-6 times/week |  |  |
| 86 | **a** | **Biscuit (salty, sweet)** | **b** | **On average how much does the child eat?** |
|  | 0 [ ] | Never | 6 [ ] | 1-2 slices (picture) |
|  | 1 [ ] | 1-2 times/month | 7 [ ] | 3-4 slices (picture) |
|  | 2 [ ] | 3-4 times/month | 8 [ ] | 5-6 slices (picture) |
|  | 3 [ ] | 1-2 times/week | 9 [ ] | 7-8 slices (picture) |
|  | 4 [ ] | 3-4 times/week | 10 [ ] | ≥ 8 slices (picture) |
|  | 5 [ ] | 5-6 times/week |  |  |
| 87 | **a** | **Ice-cream** | **b** | **On average how much does the child eat?** |
|  | 0 [ ] | Never | 6 [ ] | 1/4 portion (picture) |
|  | 1 [ ] | 1-2 times/month | 7 [ ] | 1/2 portion (picture) |
|  | 2 [ ] | 3-4 times/month | 8 [ ] | 1 portion (picture) |
|  | 3 [ ] | 1-2 times/week | 9 [ ] | 1.5 portion (picture) |
|  | 4 [ ] | 3-4 times/week | 10 [ ] | 2 portion (picture) |
|  | 5 [ ] | 5-6 times/week |  |  |
| 88 | **a** | **Soft drink (Coke, Pepsi)** | **b** | **On average how much does the child eat?** |
|  | 0 [ ] | Never | 6 [ ] | 1/4 portion (picture) |
|  | 1 [ ] | 1-2 times/month | 7 [ ] | 1/2 portion (picture) |
|  | 2 [ ] | 3-4 times/month | 8 [ ] | 1 portion (picture) |
|  | 3 [ ] | 1-2 times/week | 9 [ ] | 1.5 portion (picture) |
|  | 4 [ ] | 3-4 times/week | 10 [ ] | 2 portion (picture) |
|  | 5 [ ] | 5-6 times/week |  |  |
| 89 | **a** | **Candy, sweet** | **b** | **On average how much does the child eat?** |
|  | 0 [ ] | Never | 6 [ ] | 1/4 portion (picture) |
|  | 1 [ ] | 1-2 times/month | 7 [ ] | 1/2 portion (picture) |
|  | 2 [ ] | 3-4 times/month | 8 [ ] | 1 portion (picture) |
|  | 3 [ ] | 1-2 times/week | 9 [ ] | 1.5 portion (picture) |
|  | 4 [ ] | 3-4 times/week | 10 [ ] | 2 portion (picture) |
|  | 5 [ ] | 5-6 times/week |  |  |
